# Comparison of the efficacy of acupuncture-related Therapies for post-stroke motor aphasia: A Bayesian network meta-analysis

**DOI:** 10.3389/fneur.2022.992079

**Published:** 2022-12-20

**Authors:** Sisi Feng, Mingzhi Tang, Gan Huang, Jumei Wang, Yulan Lv, Sijin He, Duo Liu, Lihua Gu

**Affiliations:** ^1^Yunnan University of Traditional Chinese Medicine, Kunming, China; ^2^Kunming Hospital of Traditional Chinese Medicine, The Third Affiliated Hospital of Yunnan University of Chinese Medicine, Kunming, China

**Keywords:** stroke, motor aphasia, acupuncture, speech training, network meta-analysis

## Abstract

**Background:**

Motor aphasia, which can affect the communication ability of patients and even triggers severe psychological disorders, is one of the most common sequelae after stroke. Acupuncture (a typical complementary alternative therapy) is frequently combined with speech training (ST) to treat post-stroke motor aphasia (PSMA) and presents significant efficacy. However, the most effective acupuncture intervention is still unknown. This study aims to analyze the efficacy of several acupuncture approaches combined with ST for PSMA to identify the best intervention for clinical decision-making by using network meta-analysis (NMA).

**Methods:**

Eight major databases were searched from the time of their establishment to March 2022. Clinical efficacy rate (CER) was used as the primary outcome indicator. R software (version 4.13.0) and STATA software (version 16.0) were used to analyze the data.

**Results:**

A total of 29 randomized controlled trials (RCTs) and six treatment regimens were included in this study. In the pair-wise meta-analysis, we found that the efficacy of scalp-tongue acupuncture (STA) combined with ST [OR = 8.30; 95% Credible interval (CrI): 3.87, 17.33], tongue acupuncture (TA) combined with ST (OR = 3.95; 95% CrI: 2.27, 6.89), scalp-body acupuncture (SBA) combined with ST (OR = 3.75; 95% CrI: 2.26, 6.22), scalp acupuncture (SA) combined with ST (OR = 2.95; 95% CrI: 1.74, 5.0), and body acupuncture (BA) combined with ST (OR = 2.30; 95% CrI: 1.26, 4.19) were significantly superior to that of ST. In addition, the efficacy of STA + ST was significantly superior to that of SA +ST (OR = 2. 82; 95% CrI: 1.24, 6.38) and BA + ST (OR = 3.61; 95% CrI: 1.40, 9.29). According to the surface under the cumulative ranking curve (SUCRA), STA + ST (SUCRA = 97.9%) may be the best treatment regimen to improve the clinical outcome in patients with PSMA.

**Conclusion:**

The NMA showed that STA combined with ST may be the best treatment to improve CER, compared with other combination treatments. However, since the overall quality and number of studies are limited, further RCTs with a large sample and multicenter are needed for further validation.

**Systematic review registration:**

https://www.crd.york.ac.uk/prospero/display_record.php?RecordID=316081, identifier CRD42022316081.

## Introduction

Aphasia is a language impairment caused by damage to the brain's language centers (1). Stroke-induced cerebrovascular disease is the leading cause of aphasia, and it has an impact on one-third of stroke survivors. Among them, 30–43% of those affected still have language dysfunction after 6 months post-stroke (2–4). Motor aphasia, often known as Broca's aphasia, is the most common type of aphasia (5). The primary manifestation is impairment in oral expression, and even complete loss of speech, which inevitably affects the patient's ability to communicate and increases the risk of depression and other psychological disorders over time (6, 7).

Although most patients improve throughout the transition from the acute to chronic phase, persisting language impairment is still prevalent in patients (8). Currently, speech training (ST) remains the primary treatment for post-stroke aphasia. However, its effectiveness is inconsistent and restricted. A meta-analysis study of ST for post-stroke aphasia found that it is recommended that speech therapy must be provided for at least 5–10 h per week and should be started as soon as possible after a stroke. Moreover, intensive ST for over 2–3 months is essential to maximize patients' recovery from post-stroke aphasia, and failure to provide this training may affect the prognosis of patients (9). However, not all patients can bear this high intensity and frequency of training (10). Therefore, perhaps ST combined with other interventions may have a more desirable synergistic or complementary effect on the language recovery of patients (11).

As an essential part of Chinese medicine, acupuncture has a history of more than 3,000 years, and its efficacy has been widely recognized. The reconfiguration of the left brain language network has been found to be critical for language recovery in studies (12). An acupuncture study using magnetic resonance imaging (MRI) test showed that acupuncture could restore speech function by inducing activation in the most severely damaged part of the brain's left hemisphere (13). In addition, relevant clinical studies have shown that acupuncture at language-related acupoints can facilitate language recovery by promoting the reorganization of the functional cortex. All of these studies confirm the indispensable role of acupuncture in treating stroke aphasia (14). However, there are many types of acupuncture treatments for post-stroke motor aphasia (PSMA). Most clinical interventions are based on scalp, tongue, and body acupuncture (BA) and have also achieved significant efficacy. Scalp acupuncture (SA) is a method of treating the disease by stimulating acupoints or treatment areas of the cerebral cortex in the corresponding projection areas of the scalp (15). PSMA is located in the brain; thus, acupuncture of the corresponding acupoints in the head can dredge the meridians and can regulate the Qi and blood, which can promote the relative recovery of the patient's damaged speech (16). Tongue acupuncture (TA) is a newly created micro-needle therapy under the guidance of the Traditional Chinese Medicine (TCM) theory and modern biological holography. As the main vocal organ, the tongue is closely related to the internal organs and meridians. By acupuncture of specific points on the tongue body and the sublingual peripheral nerve, patients can promote their recovery of dysarthria and speech function (17). In addition, TCM theory emphasizes a holistic concept, and BA treatment is the integration of holistic and local regulation to regulate the whole body Qi activity of patients and help them restore healthy Qi and repel out pathogenic factors (18). However, it should be noted that there is a lack of guidelines to rank the efficacy of different acupuncture treatments for PSMA, which will confuse the clinical selection of appropriate treatment options for physicians. Combining direct and indirect evidence, network meta-analysis (NMA) draws on classically paired meta-analyses and summarizes the effects of many treatments for a single disease (19, 20). It can also evaluate the effectiveness of various therapies and estimate the relative efficacy of these interventions (21, 22). This study aims to examine the effectiveness of various acupuncture therapies combined with ST for treating PSMA by using NMA as the research tool, so as to provide a reference basis for selecting the best interventions for clinical application.

## Materials and methods

This NMA follows the NMA systematic review and meta-analysis preferred reporting project guidelines (23). The PROSPERO registration number for this NMA protocol is CRD42022316081.

### Search strategy

We searched the Web of Science, PubMed, EMBASE, Cochrane Central Controlled Trials, China Knowledge Network (CNKI), WanFang database, VIP database, and China Biomedical Literature Database (CBM) for randomized controlled trials (RCTs) of acupuncture combined with ST for PSMA. In addition, relevant systematic evaluations and reference lists of included studies were searched manually to ensure the comprehensiveness of included studies. The search started with the establishment of the database on March 28, 2022. The search strategy was to combine subject terms with free words (from Mesh). The subject words included: “acupuncture”, “scalp acupuncture”, “tongue acupuncture”, “electro-acupuncture”, “blood pricking therapy”, “eye-acupuncture”, “ear acupuncture”, “speech training”, “stroke”, “cerebral infarction”, “cerebral hemorrhage”, “motor aphasia”, “broca aphasia”, and “randomized controlled trials.” The search strategies such as PubMed are shown in Supplementary Table 1.

### Selection and inclusion criteria

The inclusion criteria were as follows. (1) According to the clear diagnostic criteria, regardless of gender and age, patients were diagnosed with PSMA. However, the article should clearly describe that the baseline was comparable between the groups (*P* > 0.5). (2) RCTs. (3) The treatment group received ST combined with different acupuncture therapies [e.g., SA, TA, BA, scalp-tongue acupuncture (STA), and scalp-body acupuncture (SBA)]. There is no restriction on acupuncture point selection, stimulation intensity, or treatment mode. The control group was treated with ST alone or intercomparison between interventions. (4) The primary outcome was the clinical effective rate (CER). Based on the presence of clinical symptoms and objective indicators, efficacy was divided into valid and invalid categories. No improvement in clinical symptoms was considered invalid. CER = (total number – invalid number)/total number × 100% (24).

Exclusion criteria included: (1) The research subjects were non-clinical patients; (2) Participants did not meet the inclusion criteria, such as non-PSMA; (3) outcome indicators that failed to meet the inclusion criteria were included; (4) The treatment group received non-acupuncture therapy combined with ST; (5) repetitively published studies; and (6) Unavailability of full-text studies.

### Study selection and data extraction

EndNote version 20 was used to exclude duplicate literature, and the first round of screening was performed by reading the titles and abstracts. Literature that did not meet the inclusion criteria was excluded by re-evaluating the whole text. The data were extracted by two researchers (MT and GH). In the event of a disagreement, the third researcher (SH) would make the final decision. Title, author, publication year, sample size, age, disease course, treatment group, control group interventions, duration of treatment, and outcome indicators were included in the data extraction contents.

### Risk of bias assessment

Both JMW and YLL used the RCT risk of bias assessment tool of the Cochrane Handbook of Systematic Reviews version 5.1.0 (25) to assess the overall quality of the studies, and a third investigator (DL) assisted them in determining the degree of variation in their findings. The assessment consisted of seven items, and each item was rated as low, medium, or high risk of bias. Review Manager version 5.4 was used to generate the risk of bias data (Cochrane, London, UK).

### Statistical analysis

Network meta-analysis was performed using R software version 4.13.0 based on the Bayesian framework of the Markov chain Monte Carlo (MCMC) consistency model. The initial value was set using four Markov chains. The number of iterations for the first update of the model was set to 50,000, and the number of iterations for continuous updates was set to 100,000. To eliminate the effect of the initial value, the first 50,000 anneals were discarded and the sampling was started from 50,001 iterations. Estimation and extrapolation assume that the density reaches a steady state under which the convergence of the results is evaluated using the potential scale reduction parameter (PSRF). Convergence is suggested to be complete when the PSRF is between 1 and 1.5, indicating that the model is stable enough for data analysis. Dichotomous variables were expressed as the ratio of odds ratios (ORs) and 95% confidence intervals (CIs).

Stata software version 16.0 (StataCorp, College Station, TX, USA) was used to draw a network evidence map to show the close relationship between interventions and each outcome indicator. The loop inconsistency test assessed the inconsistency between direct and indirect comparisons in the presence of closed loops. When the inconsistency factor (IF) was close to zero, 95% CI included zero. It indicated that the direct evidence was consistent with the indirect evidence and the results were reliable. The cumulative ranking curve [surface under the cumulative ranking curve (SUCRA)] was used to rank the probability of different interventions; the higher the scores of SUCRA, the better the efficacy or safety. Minor sample effects or publication bias were detected by comparing corrected funnel plots.

## Results

### Characteristics of the included studies

**Figure 1:**
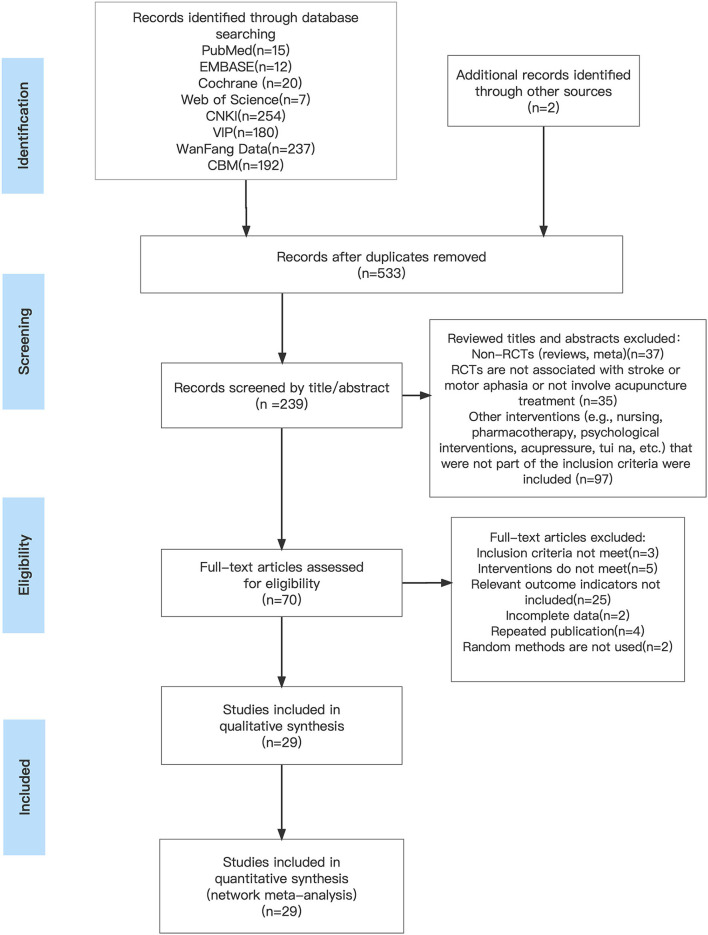
Literature screening process.

A total of 919 studies were searched. After several rounds of screening, 29 RCTs were finally included (26–54), and 2,082 patients were included in the total. Figure 1 depicts the process of screening the literature.

Of these, 28 trials (26–44, 46–54) were two-armed RCTs, and 1 trial (45) was three-armed. A total of six interventions were also included, such as ST, STA combined with ST, TA combined with ST, SBA combined with ST, SA combined with ST, and BA combined with ST. Notably, all 29 studies reported CER. Specific baseline details are shown in Table 1.

**Table 1:** Characteristics of included studies.

**Study ID**	**Participant**			**Age**	**Gender (M/F)**	**Interventions**			**Course**
	**T**	**T1**	**C**			**T**	**T2**	**C**	
Qin (26)	33		32	T: 60.52 ± 5.89	T: 23/10	BA + ST		ST	6w
				C: 58.44 ± 6.81	C: 28/ 4				
Fan (28)	34		34	T: 57.85 ± 8.85	T: 18/16	TA + ST		ST	2w
				C: 57.21 ± 9.08	C: 20/14				
Yu (27)	30		30	T: 62.80 ± 9.92	T: 17/13	SBA + ST		ST	8w
				C: 65.40 ± 9.45	C: 19/11				
Wang et al. (31)	35		35	T: 65.4 ± 4.6	T: 19/16	STA + ST		SA +ST	4w
				C: 64.5 ± 4.8	C: 20/15				
Ren et al. (33)	35		35	T: 53.0 ± 4.0	T: 19/16	TA + ST		ST	4w
				C: 55.0 ± 6.0	C: 15/20				
Li et al. (32)	51		51	T: 62.08 ± 7.11	T: 28/23	TA + ST		BA +ST	12w
				C: 63.42 ± 6.77	C: 24/27				
Xu (30)	25		25	T: 64.44 ± 9.7	T: 14/11	TA + ST		ST	8w
				C: 64.72 ± 7.87	C: 15/10				
Zhang (29)	25		24	T: 63.40 ± 8.28	T: 15/10	BA + ST		ST	4w
				C: 59.33 ± 8.13	C: 20/ 4				
Zhang and Sun (34)	38		38	T: 57.32 ± 7.43	T: 21/17	SA + ST		ST	8w
				C: 59.16 ± 7.9	C: 23/15				
Yin (35)	40		40	T: 59.70 ± 12.4	T: 27/13	SBA + ST		ST	4w
				C: 59.48 ± 10.9	C: 29/11				
Yang et al. (36)	30		30	T: 65 ± 9.66	T: 18/12	STA + ST		SBA + ST	3w
				C: 61.97 ± 13.2	C: 21/ 9				
Quan (37)	30		30	T: 56.77 ± 10.1	T: 18/12	SBA + ST		SA + ST	4w
				C: 57.73 ± 9.82	C: 17/13				
Jin et al. (37)	40		39	T: 53.98 ± 9.46	T: 23/17	SA + ST		BA + ST	4w
				C: 54.60 ± 7.65	C: 21/18				
Teng and Hong (39)	46		45	T: 56.30 ± 17.4	T: 24/22	SA + ST		ST	30d
				C: 57.60 ± 16.5	C: 25/20				
Xiong et al. (40)	32		32	T: 63.58 ± 7.44	T: 20/12	BA + ST		ST	5w
				C: 64.08 ± 7.67	C: 18/14				
Wang (41)	30		30	T: 58.24 ± 8.27	T: 18/12	SA + ST		ST	4w
				C: 59.10 ± 8.88	C: 16/14				
He et al. (42)	40		40	T: 53.86 ± 7.12	T: 26/14	STA + ST		ST	8w
				C: 54.18 ± 6.25	C: 25/15				
Hu (43)	30		30	T: 65.33 ± 9.33	T: 17/13	TA + ST		ST	30d
				C: 66.10 ± 6.70	C: 20/10				
Zhang (44)	30		30	T: -	T: 20/10	SA + ST		ST	3w
				C: -	C: 22/ 8				
Hou et al. (45)	30	30	30	T: 57.07 ± 10.9	T:16/14	SA + ST	SB +ST	ST	14d
				T2: 57.10 ± 11	T2:13/17				
				C: 56.70 ± 10.5	C:15/15				
Li and Yue (46)	36		31	T: 65.16 ± 8.09	T: 21/15	TA + ST		ST	15d
				C: 62.45 ± 7.92	C: 19/12				
Gu (47)	43		41	T: 62.80 ± 7.12	T: 23/15	TA + ST		ST	30d
				C: 63.69 ± 6.79	C: 22/14				
Huang and Huang (48)	42		42	T/C : 62 ± 5.0	T: 22/20	BA + ST		ST	2w
					C: 21/21				
Chen (50)	30		30	T: 61.38 ± 4.23	T: 17/13	STA + ST		ST	60d
				C: 61.25 ± 4.14	C: 16/14				
Huang (49)	58		57	T: 65.85 ± 3.02	T: 31/27	SBA + ST		ST	28d
				C: 64.86 ± 5.39	C: 29/28				
Liang (52)	39		39	T: 65.13 ± 11.32	T: 28/11	STA + ST		BA + ST	2w
				C: 66.44 ± 10.41	C: 29/10				
Shen and Shao (54)	20		20	T: 50.40 ± 11.87	T: 12/8	STA + ST		ST	8w
				C: 54.70 ± 8.63	C: 9/11				
Piao (53)	30		30	T: 58.20 ± 7.31	T: 15/15	SBA + ST		ST	30d
				C: 58.43 ± 6.85	C: 13/17				
Huang (49)	48		48	T: 60.96 ± 9.46	T: 24/24	SA + ST		ST	30d
				C: /	C: 26/22				

BA, body acupuncture; SA, scalp acupuncture; TA, tongue acupuncture; SBA, scalp-body acupuncture; STA, scalp-tongue acupuncture; ST, speech training; C, control group; T, treatment group; m, month; d, day; w, week; CER, clinical effective rate.

### Quality evaluation

Regarding random sequence generation, 17 studies (26, 28, 31, 33–37, 41, 42, 45, 47–49, 51, 53, 54) used random number tables, seven studies (29, 38, 43, 44, 46, 50, 52) used computer-generated random numbers, and 5 studies (27, 30, 32, 39, 40) mentioned randomness only. Regarding distribution concealment, five studies (33, 38, 44, 47, 52) used opaque envelopes, while the remaining 21 trials did not unclear it. Due to the limitations of the intervention, just two studies (29, 46) proposed the blinding of investigators. In four studies (29, 38, 46, 47), investigators were masked to the outcome indicators assessors. A total of 26 studies (26–54) reported prespecified outcome indicators. Moreover, five studies (26, 28, 29, 34, 44) mentioned safety. The results are shown in Supplementary Figures 1, 2.

### Network meta-analysis

#### Clinical effectiveness rate

**Figure 2:**
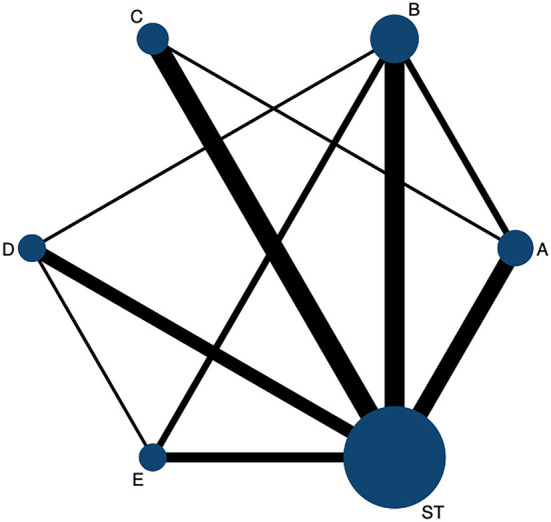
The network evidence graph for CER. ST, Speech training; BA, body acupuncture; SA, scalp acupuncture; TA, tongue acupuncture; SBA, scalp-body acupuncture; STA, scalp-tongue acupuncture.

A total of 29 studies reported the CER (26–54), including 10 direct comparisons [ST vs. STA + ST (*n* = 3), ST vs. SBA + ST (*n* = 4), ST vs. TA (*n* = 6), ST vs. SA + ST (*n* = 6), ST vs. BA + ST (*n* = 5), BA + ST vs. SA + ST (*n* = 2), BA + ST vs. TA + ST (*n* = 1), SA + ST vs. SBA + ST (*n* = 1), SA + ST vs. STA + ST (*n* = 2), SBA + ST vs. STA + ST (*n* = 1)]. Figure 2 shows the network evidence graph. The results of NMA of clinical efficacy are shown in Table 2. Compared with ST alone, STA + ST (OR = 3.80; 95% CrI: 3.87, 17.83), TA + ST (OR = 3.95; 95% CrI: 2.27, 6.89), SBA + ST (OR = 3.75; 95% CrI: 2.26, 6.22), SA + ST (OR = 2.95; 95% CrI: 1.74, 5.00), and BA + ST (OR = 2.30; 95% CrI: 1.26, 4.19) were all related to the improvement of CER. In addition, the efficacy of STA + ST was significantly superior to that of SA +ST (OR = 3.61; 95% CrI: 1.40, 9.29) and BA + ST (OR = 2.82; 95% CrI: 1.24, 6.38).

**Table 2:** Relative effect sizes of CER efficacy after the intervention.

SUCRA 97.8%					
**E**	SUCRA 66.8%				
2.10 (0.82, 5.37)	**C**	SUCRA 62.6%			
2.21 (0.94, 5.19)	1.05 (0.50, 2.22)	**D**	SUCRA 47.8%		
**2.82** **(1.24, 6.38)**	1.34 (0.63, 2.84)	1.27 (0.66, 2.46)	**B**	SUCRA 26.1%	
**3.61** **(1.40, 9.29)**	1.72 (0.83, 3.55)	1.63 (0.76, 3.52)	1.28 (0.63, 2.60)	**A**	SUCRA 0.1%
**8.30** **(3.87, 17.83)**	**3.95 (2.27, 6.89)**	**3.75** **(2.26, 6.22)**	**2.95 (1.74, 5.00)**	**2.30** **(1.26, 4.19)**	**ST**

Treatments were ranked in order of their likelihood of being the best treatment. The above data represent the confidence interval. The bold font indicates that there was a statistically significant difference between the two treatments. BA, body acupuncture; SA, scalp acupuncture; TA, tongue acupuncture; SBA, scalp-body acupuncture; STA, scalp-tongue acupuncture; ST, speech training; CER, clinical effective rate; SUCRA, The surface under the cumulative ranking curve.

According to the results of the SUCRA probability ranking chart (Table 2 and Supplementary Figure 3), STA + ST was probably the most effective intervention to improve clinical efficacy (97.8%), followed by TA + ST (66.8%). The 6 interventions are ranked as follows: STA + ST (97.8%) > TA + ST (66.8%) > SBA + ST (62.6%) > SA + ST (44.3%) > BA + ST (28.3%) > ST (0.1%).

**Figure 3:**
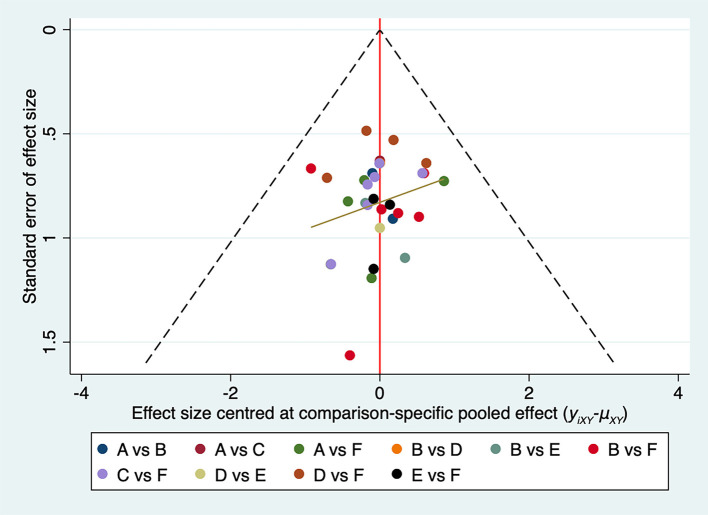
Comparison-adjusted funnel plots for the CER network. ST, Speech training; BA, body acupuncture; SA, scalp acupuncture; TA, tongue acupuncture; SBA, scalp-body acupuncture; STA, scalp-tongue acupuncture.

We plotted a comparison-adjusted funnel plot for the CER. As shown in Figure 3, studies were symmetrical in distribution on both sides of the inverted funnel; however, there was still a scattered point below the inverted funnel. Therefore, we further performed Egger's test, and the result showed *P* = 0.327 (>0.05), suggesting that the possibility of risk of bias in this study is low (Supplementary Table 2).

#### Model convergence and consistency test

Inconsistency can affect the accuracy of NMA and threaten the validity of the results. Thus, consistency testing is an essential part of the NMA process, and it provides specific assurance for the reliability of indirect evidence with the available statistical efficiency (55, 56). This study showed that the network analysis of the primary outcome was consistent with the consistency model (*P* = 0.514; >0.05). In the Brooks Gelman Rubin diagnostic plots, all of the shrinking factors were < 1.2 (Supplementary Figure 4). The IF values of each closed loop were distributed from 0.10 to 1.29 and the lower limit of 95% CI contained 0 (Supplementary Figure 5). Clearly, the results show no apparent signs of inconsistency in this NMA.

## Discussion

To the best of our knowledge, this is the first Bayesian NMA study using different acupuncture techniques combined with ST for PSMA. From the 29 RCTs (26–54), we included five acupuncture therapies commonly used in the clinical treatment of PSMA combined with ST, BA + RT, SA + RT, TA + RT, SBA + RT, and STA+ RT, with significant efficacy compared with ST.

Post-stroke motor aphasia is a disease that severely affects the communication ability of patients and even triggers a range of psychological disorders. Thus, it is urgent to explore a safe and effective treatment option. As an alternative therapy for post-stroke aphasia rehabilitation, acupuncture has some typical advantages (57), such as no side effects, ease of operation, low cost, and high compliance. Also, it is more acceptable to most patients. A mechanistic study on acupoint stimulation pointed out that after nerve injury, long-term intense stimulation of acupoints will produce an inflammatory response, sensitize receptors and ion channels on local nerve fibers, and stimulate relevant receptors on acupoints to converge on spinal horn neurons so as to regulate body functions (58). Among the five different acupuncture techniques combined with ST interventions, STA combined with ST may be the most potential one for treating PSMA. This study showed that STA + ST (OR = 3.80; 95% CrI: 3.87, 17.83; SUCRA = 97.8%) was the optimal treatment option to improve the clinical efficiency rate in patients with PSMA.

According to Chinese medicine, the head is closely related to the five Zang organs. Precisely because the disease location of PSMA is in the brain, through the acupuncture of head acupoints, it can stimulate the meridians and internal organs that are connected to it, dredging the meridian, opening the orifice, and producing sound. Modern medical research on cerebral blood flow, EEG, and blood rheology pointed out that acupuncture of scalp points can improve cerebral blood circulation, increase blood flow, restore blood supply to diseased brain tissue, and improve cerebral electrical activity and cortical inhibition, thus activating brain speech function (59, 60). The clinical treatment of PSMA is based chiefly on acupuncture in the speech I region, namely, the projection of Broca's area on the scalp. Coincidentally, the speech I region coincides with the somatotopic projections of mirror neuron brain regions. Also, the activation of the mirror neuron system has an actual language support function, and it is involved in language processing and plays a significant role in activating language response mechanisms (61). Related studies have shown that stimulation of dominant hemispheric lesions and perifocal areas (Broca's area) along with the activation of nondominant hemispheric mirror areas (which coincide with language areas) leads to the strengthening of central reflex connections in language and contributes to the activation and neural remodeling of brain language networks (62, 63). However, SA + ST (SUCRA = 44.3%) ranked relatively weakly in our study results in terms of improved clinical effectiveness. Moreover, the efficacy of the combined treatment of STA + ST (OR = 3.61; 95% CrI: 1.40, 9.29) was significantly superior to that of SA + ST.

Thus, it is noteworthy that as an essential organ of phonation, the tongue plays an essential role in speech rehabilitation. It is closely related to the heart, spleen, kidney, and other visceral organs. Stimulating the acupuncture points on the tongue can dredge the blood stasis meridians on the tongue, improve the local blood supply, and increase the elasticity of the tongue. Also, it helps to regulate the Qi and blood to nourish the tongue's body, thus facilitating the recovery of speech function (43). Modern medicine believes that abundant nerve tissues are distributed on the tongue body, and the stimulation of acupuncture of tongue roots can reflexively enhance the excitability of the central nervous system. Balancing specific and nonspecific conduction systems through cortical-thalamo-cortical regulation can further reconstruct the neural circuit of speech activity and accelerate the recovery of speech function (64). Due to the inconvenience of leaving the needle in the tongue, blood pricking therapy, acupuncture of Jinjin (EX-HN12), Yuye (EX-HN13), and tongue points corresponding to the heart, spleen, and kidney are frequently adopted clinically (65). Studies on related mechanisms pointed out that the anatomical sites of the heart, spleen, kidney, EX-HN12, and EX-HN13 points are between the thyroid cartilage and the root of the tongue and have pharyngeal, vagus, sublingual, and hyoid muscle nerve distribution with the ability to innervate the pharyngeal muscles and vocal cords. Acupuncture of these points stimulates the lingual root nerve, activates reflex pathways, strengthens excitatory reflexes, and promotes recovery of damaged and deformed neurons, thus improving motor aphasia (66). Our study is generally consistent with the above findings that TA + ST (SUCRA = 66.8%) is the second most crucial intervention after STA + ST (SUCRA = 66.8%) for the treatment of PSMA. However, according to TCM, language production is a joint effort between the heart (brain) and the tongue. Although TA has advantages, interventions combined with STA can provide significantly better efficacy. Thus, it is more worthy of clinical application.

Notably, BA + ST (SUCRA = 28.3%) ranked the last among five acupuncture interventions. The concept of BA treatment is based on the holistic concept of Chinese medicine. TCM believes that language is a manifestation of consciousness, and BA treatment, based on the “Xingnao Kaiqiao” acupuncture method, achieves the purpose of opening the linguae orifices by means of inducing resuscitation and harmonizing the Qi and blood (67). The function mechanism in the treatment of PSMA may be related to the following: (1) improving blood rheology, increasing cerebral oxygen supply and blood flow, and significantly reducing brain tissue necrosis; (2) improving brain electrical activity and stimulating brain language function; and (3) improving the metabolism of ATPase in microvascular endothelial cells, thus promoting brain metabolism (40). However, two clinical studies on PSMA (68) pointed out that considering the specificity of post-stroke aphasia regarding disease location, “Xingnao Kaiqiao” acupuncture may lack targeting for language function improvement. This may explain why the efficacy of BA is not superior in treating PSMA compared to other acupuncture interventions. However, it still needs to be explored by including more direct evidence in the future.

### Limitations

However, there are still some limitations to our study. First, all of the studies that were included were from China. No studies from other countries may make the results less accurate and convincing. Second, although no significant inconsistencies were found in this NMA, considering the small sample size of individual studies, the small number of included studies, and the lack of more direct comparisons between acupuncture treatments, it may lead to some potential bias and affect the reliability of the results. Finally, given the relatively small number of patients included, additional multicenter and high-quality RCTs are still needed to validate our findings in the future.

## Conclusion

After a thorough comparison of the effectiveness indicators of six different treatments, the Bayesian NMA showed that scalp and TA combined with ST (STA + ST) may be the best acupuncture-related therapies to improve clinical outcomes in patients with motor aphasia after stroke. Since the current literature is limited and some reports are of average quality, more controlled trials with large samples and multicenter are necessary to validate the available evidence. However, our study still provides reliable information for PSMA treatment decision-making. Also, it is still recommended that physicians make reasonable choices in clinical practice based on the specific situation of their patients.

## Data availability statement

The original contributions presented in the study are included in the article/Supplementary material, further inquiries can be directed to the corresponding author.

## Author contributions

SF designed the study and wrote the manuscript. MT, GH, JW, and YL participated in the extraction and analysis of the data. LG, SH, and DL critically supervised, evaluated, and validated the article. All of the authors worked on the article and agreed with the submitted version.

## Supplementary material

Supplementary Figure 1 Quality assessment percentage graph.

Supplementary Figure 2 Summary chart of quality assessment.

Supplementary Figure 3 Probability ranking results of CER of different interventions. ST, Speech training; BA, body acupuncture; SA, scalp acupuncture; TA, tongue acupuncture; SBA, scalp-body acupuncture; STA, scalp-tongue acupuncture.

Supplementary Figure 4 PSRF diagnostic diagram for CER.

Supplementary Figure 5 Loop inconsistency tests. ST, Speech training; BA, body acupuncture; SA, scalp acupuncture; TA, tongue acupuncture; SBA, scalp-body acupuncture; STA, scalp-tongue acupuncture.

## References

[1] National Institute on Neurological Disorders and Stroke. Aphasia Hope Through Research. Bethesda, MD: National Institute on Neurological Disorders and Stroke (1990).

[2] Norise C Hamilton RH. Non-invasive brain stimulation in the treatment of post-stroke and neurodegenerative aphasia: parallels, differences, and lessons learned. Front Hum Neurosci. (2016) 10:675. 10.3389/fnhum.2016.00675 28167904 PMC5253356

[3] Hamilton RH. Neuroplasticity in the language system: reorganization in post-stroke aphasia and in neuromodulation interventions. Restor Neurol Neurosci. (2016) 34:467–71. 10.3233/RNN-169002 27472848

[4] Hillis AE Work M Barker PB Jacobs MA Breese EL Maurer K. Re-examining the brain regions crucial for orchestrating speech articulation. Brain. (2004) 127:1479–87. 10.1093/brain/awh172 15090478

[5] Lv YM. Common methods of rehabilitation for motor aphasia due to cerebrovascular disease. J Med Univ. (2006) 27:75–7.

[6] Liu Y Mao SP Zhu HF Shen J Zhang YP Li ZZ. Influencing factors of psychological disorders of patients with aphasia after stroke and lust of homeopathic therapy. China J Health Psychol. (2013) 21:1653–5.

[7] Lindsay LR Lercher K O'Dell MW. Should this patient with global aphasia after a left cerebral stroke be admitted to your hospital-based inpatient rehabilitation unit? PMR. (2017) 9:629–35. 10.1016/j.pmrj.2017.05.002 28602175

[8] Wade DT Hewer RL David RM Enderby PM. Aphasia after stroke: natural history and associated deficits. J Neurol Neurosurg Psychiatry. (1986) 49:11–6. 10.1136/jnnp.49.1.11 2420939 PMC1028640

[9] Bhogal SK Teasell R Speechley M. Intensity of aphasia therapy, impact on recovery. Stroke. (2003) 34:987–93. 10.1161/01.STR.0000062343.64383.D0 12649521

[10] Koyuncu E Çam P Altinok N Çalli DE Duman TY. Özgirgin N. Speech and language therapy for aphasia following subacute stroke. Neural Regen Res. (2016) 11:1591–4. 10.4103/1673-5374.193237 27904489 PMC5116837

[11] Brady MC Godwin J Kelly H Enderby P Elders A. Campbell P. Attention control comparisons with SLT for people with aphasia following stroke: methodological concerns raised following a systematic review. Clin Rehabil. (2018) 32:1383–95. 10.1177/0269215518780487 29911416

[12] Rafols JA. Control of the brain microcirculation following traumatic brain injury and stroke. Brain Circ. (2015) 1:146–58. 10.4103/2394-8108.172892

[13] Chang J Zhang H Tan Z Xiao J Li S Gao Y. Effect of electroacupuncture in patients with post-stroke motor aphasia: neurolinguistic and neuroimaging characteristics. Wien Klin Wochenschr. (2017) 129:102–9. 10.1007/s00508-016-1070-1 27590260

[14] Li G. Yang ES. An fMRI study of acupuncture-induced brain activation of aphasia stroke patients. Complement Ther Med. (2011) 19:S49–59. 10.1016/j.ctim.2010.11.004 21195295

[15] Wu JL Yin HN Wang DL Zhu ZW Sun ZR. Origin and development situation of scalp acupuncture therapy. J Univ Trad Chin Med. (2019) 36:1783–7.

[16] Lou XQ Liu X Liu CH Lin HJ Liu H Ling J. Therapeutic effect of electric-balance stimulation with scalp acupuncture for motor aphasia after cerebral infarction. Chin Acupunct Moxib. (2021) 41:1211–5. 34762372 10.13703/j.0255-2930.20210302-k0005

[17] Wang TR Liu Q Zhao LX Gao Y Zhao BX. Clinical observations on tongue acupuncture combined with language therapy in motor aphasia after stroke. Shandong J Trad Chin Med. (2016) 35:36–7.

[18] Yu J Sun ZR Li HL Chang WZ Wang Y. Reflection on the discussion aiming at acupuncture, healthy qi of traditional Chinese medicine and modern immunology. China Med Herald. (2015) 12:77–80.

[19] Grant RL. The uptake of Bayesian methods in biomedical meta-analyses: a scoping review (2005–2016). J Evid Based Med. (2019) 12:69–75. 10.1111/jebm.12326 30511364

[20] Zhang D Wu J Duan X Wang K Ni M Liu S . Network meta-analysis of chinese herbal injections plus the FOLFOX regimen for the treatment of colorectal cancer in China. Integr Cancer Ther. (2019) 18:1534735419827098. 10.1177/1534735419827098 30791732 PMC7242776

[21] Greco T Biondi-Zoccai G Saleh O Pasin L Cabrini L Zangrillo A . The attractiveness of network meta-analysis: a comprehensive systematic and narrative review. Heart Lung Vessel. (2015) 7:133–42. 26157739 PMC4476767

[22] Rouse B Chaimani A Li T. Network meta-analysis: an introduction for clinicians. Intern Emerg Med. (2017) 12:103–11. 10.1007/s11739-016-1583-7 27913917 PMC5247317

[23] Hutton B Salanti G Caldwell DM Chaimani A Schmid CH Cameron C. et al. The PRISMA extension statement for reporting of systematic reviews incorporating network meta-analyses of health care interventions: checklist and explanations. Ann Intern Med. (2015) 162:777–84. 10.7326/M14-2385 26030634

[24] Ministry of Health of People's Republic of China. Guidelines of TCM Clinical Research, 1st Edn. Beijing: People's Health Publishing House (1993).

[25] Savović J Weeks L Sterne JA Turner L Altman DG Moher D . Evaluation of the Cochrane Collaboration's tool for assessing the risk of bias in randomized trials: focus groups, online survey, proposed recommendations and their implementation. Syst Rev. (2014) 3:37. 10.1186/2046-4053-3-37 24731537 PMC4022341

[26] Qin MN. The *Clinical Observation of Xingnao-Kaiqiao Acupuncture Combined with Language Rehabilitation Training on Motor Aphasia after Cerebral Infarction*. Tianjin: Tianjin University of Traditional Chinese Medicine (2021).

[27] Yu L. Clinical Observation of Acupuncture Combined with Speech Rehabilitation Training in the Treatment of Motor Aphasia after Apoplexy. Anhui: Anhui University of Chinese Medicine. (2021).

[28] Fan MD. Clinical Study of Tongue Acupuncture Combined With Language Rehabilitation Training in the Treatment of Motor Aphasia After Stroke. Yunnan: Yunnan University of Chinese Medicine (2021).

[29] Zhang YF. The Clinical Observation of Xingnao-Kaiqiao Acupuncture Combined with Language Rehabilitation Training on Motor Aphasia after Cerebral Infarction. Tianjin: Tianjin University of Traditional Chinese Medicine (2020).

[30] Xu HQ Chen J. Clinical observation on tongue acupuncture for improving various speech ability of patients with motor aphasia. Clin Res. (2020) 28:103–4.

[31] Wang LS Li J Gu XT Li GN He YG. Efficacy of tongue and head acupuncture with speech training in the treatment of motor aphasia after ischemic stroke. Hebei J Trad Chin Med. (2020) 42:118–21.

[32] Li WQ Yu J Wang CY Jin N Cheng XL. Clinical Study of Tongqiao Kaiyin acupuncture combined with language rehabilitation training on post-stroke motor aphasia. J Clin Acupunct Moxib. (2020) 36:16–20.

[33] Ren CZ Lv XY Zhang XJ Zhang XH Zhang HT. Acupuncture technique of Jingou Diaoyu combined with speech rehabilitation training for post-stroke motor aphasia. Chin Acupunct Moxib. (2020) 40:1037–41. 33068342 10.13703/j.0255-2930.20190811-k0002

[34] Zhang RM Sun SB. Treatment of motor aphasia after stroke with scalp acupuncture and speech rehabilitation. J Univ Chin Med. (2020) 36:339–42.

[35] Yin DX. Clinical Efficacy of Acupuncture With Language Rehabilitation Training in the Treatment of Post-Stroke Motor Aphasia. Liaoning: Liaoning University Of Traditional Chinese Medicine (2020).

[36] Yang Y Liu GX Xu Y Zhou T Li J. Clinical effect of acupuncture at “the five acupoints for improving aphasia” combined with language rehabilitation training in treatment of motor aphasia after ischemic stroke. J Univ Chin Med. (2019) 38:48–51.

[37] Quan LL. Clinical Observation on the Treatment of Post-Stroke Motor Aphasia by Acupuncture of Tongue Three Acupuncture Points as the Main Acupoints. Liaoning: Liaoning University of Traditional Chinese Medicine. (2019).

[38] Jin RX Zhang C Ni HL Zhou F. Effects of scalp acupunture combined with language rehabilitation training on patients with motor aphasia after stroke. J Med Univ. (2018) 41:1503–7. 25044172

[39] Teng YY Hong J. Clinical observation of scalp acupuncture plus speech rehabilitation for Broca's aphasia after cerebral stroke. J Acupunct Tuina Sci. (2017) 15:104–8. 10.1007/s11726-017-0984-0

[40] Xiong J Zhang M Guo WL Fan SH Zhang YL. Clinical research on Xingnao Kaiqiao acupuncture method combined with language rehabilitation training in patients with cerebral infarction motor aphasia. Acta Chinese Medicine. (2016) 31:1609–13.

[41] Wang N. The Clinical Effects on the Cluster Scalpacupuncture Plus Language Rehabilitation Training around the Anterior Oblique Line of Vertex-temporal for Motor Aphasia after Cerebal Infarction. Heilongjiang: Heilongjiang University of Chinese Medicine. (2015).

[42] He JF Xu XH Mao HX Wang LD Chen H Wang WM. Clinical observation of rehabilitation therapy combined with acupuncture in treatment of motor aphasia after stroke. Chin Archiv Trad Chin Med. (2015) 33:763–5.

[43] Hu ME. Clinical Research of Therapeutical Effect of Tongue Acupuncture with the Language of Rehabilitation Training on Broca Aphasia Following Stroke. Guangzhou: Guangzhou University of Chinese Medicine (2013).

[44] Zhang XF. Clinical Research on Motor Aphasia After Stroke With Jin's Scalp Acupuncture and Speech Therapy. Guangzhou: Guangzhou University of Chinese Medicine (2012).

[45] Hou WH Chang DH Yang CX Shi GC Zhou L. The efficacy of head electroacupuncture in the treatment of motor aphasia after stroke. J Clin Acupunct Moxib. (2012) 28:29–31.

[46] Li L Yue ZH. Blood-letting therapy combined with speech rehabilitation in the treatment of motor aphasia after stroke. J Clin Acupunct Moxib. (2011) 27:24–6.

[47] Gu YC. Clinical Research on Acupuncture in Jinjinyuye Point with Speech Therapy for Broca Aphasia Following the Stroke. Fujian: Fujian University of Traditional Chinese Medicine (2009).

[48] Huang SZ Huang K. Effect of acupuncture with speech training on speech function recovery of aphasia patients after stroke. World Chin Med. (2016) 11:1074–6. 25044172

[49] Huang HY. Effect of scalp acupuncture combined with speech therapy on ischemic stroke. Chin J Rehab Theor Prac. (2009) 15:1180–2.

[50] Chen LL Lin WQ Li XY Lin X. Study on the effect of acupuncture combined with rehabilitation on speech function of patients with motor aphasia after stroke. Shenzhen J Int Trad Chin Western Med. (2022) 32:10–4.

[51] Huang QS. Effect of acupuncture combined with speech rehabilitation in patients with motor aphasia after ischemic stroke. Clin Res. (2022) 30:136–9.

[52] Liang XJ. The Efficacy of Toning Acupuncture in the Treatment of Motor Aphasia After Ischemic Stroke. Guangzhou: Guangzhou University of Chinese Medicine (2021).

[53] Piao ZS. Clinical Study of Acupuncture Combined With Language Rehabilitation Training for the Treatment of Motor Aphasia After Stroke. Changchun: Changchun University of Chinese Medicine (2016).

[54] Shen XS Shao J Li B. Clinical observation on 20 cases of aphemia after ischemic cerebral apoplexy treated by acupuncture combined with language rehabilitation. J Gansu Univ Chin Med. (2019) 36:62–6.

[55] Donegan S Williamson P D'Alessandro U Tudur Smith C. Assessing key assumptions of network meta-analysis: a review of methods. Res Synth Methods. (2013) 4:291–323. 10.1002/jrsm.1085 26053945

[56] Zhang C Yan JZ Sun F Liu Q Guo Y Zeng XT. Differentiation and handling of homegeneity in network meta-analysis. Chin J Evid Based Med. (2014) 14:884–8.

[57] Tang J Zhang H Han GD Ai K Deng SF. Acupuncture for aphasia:a retrospective analysis of clinical literature. Chin Acupunct Moxibu. (2016) 36:431–6. 27352512

[58] Zhao JJ Li SY Rong PJ Ben H Zhu B. Investigation on effects and mechanism of acupoints. Modern Trad Chin Med Mat Med World Sci Technol. (2014) 16:2076–82.

[59] Fei AH Cai SC Xu B. Clinical research of post-stroke motor aphasia treated with acupoint application of jieyu plaster combined with acupuncture. Chin Acupunct Moxib. (2015) 35:1099–102. 26939316

[60] Jiang HH Zhou YL Liang XP Hong WJ Lin L Zheng HB. Integrative analysis of clinical efficacy in patients with motor aphasia and its impact on the quality of the language of daily life after stroke. Chin Archiv Trad Chin Med. (2015) 33:1235–7.

[61] Geranmayeh F Brownsett SL Wise RJ. Task-induced brain activity in aphasic stroke patients: what is driving recovery? Brain. (2014) 137:2632–48. 10.1093/brain/awu163 24974382 PMC4163030

[62] Evelina F Sharon L Thompson S. Reworking the language network. Trends Cognit Sci. (2014) 18:120–6. 10.1016/j.tics.2013.12.006 24440115 PMC4091770

[63] Marangolo P Fiori V Calpagnano MA Campana S Razzano C Caltagirone C . tDCS over the left inferior frontal cortex improves speech production in aphasia. Front Hum Neurosci. (2013) 7:539. 10.3389/fnhum.2013.00539 24046740 PMC3764371

[64] Hu XW. Clinical Research on Acupuncture in “Wu Quan” Point With Language Training for broca aphasia Following the Stroke. Henan: Henan University of Chinese Medicine (2010).

[65] Guan ZH Guan WW Guan AR Ding LL Li Q Wang ZH . Origin and clinical applications of GUANs' tongue needling techniques. China J Trad Chin Med Pharm. (2021) 36:6546–50.

[66] Guan AR Guan WW Li Q Ding LL Wang SN Guan ZH. Exploration of the mechanism of therapeutic action of tongue acupuncture. Lishizhen Med Mat Med Res. (2016) 27:914–5.

[67] Liu Y Wang YQ. “Xingnaokaiqiao” acupuncture combined with clinical observation on treatment of 100 cases of apoplectic aphasia rehabilitation therapy. J Clin Exp Med. (2013) 12:637–8.

[68] Sun XR. Analysis and Research on Curative Effect and Influencing Factors of Acupuncture on Aphasia After Acute Ischemic Stroke. Tianjin: Tianjin University of Traditional Chinese Medicine (2020).

