# Supplementary figures and images for: Comparison of the efficacy of acupuncture-related Therapies for post-stroke motor aphasia: A Bayesian network meta-analysis
Source: Front Neurol. 2022 Dec 20;13:992079. doi: 10.3389/fneur.2022.992079 (PMC9810494; doi:10.3389/fneur.2022.992079)

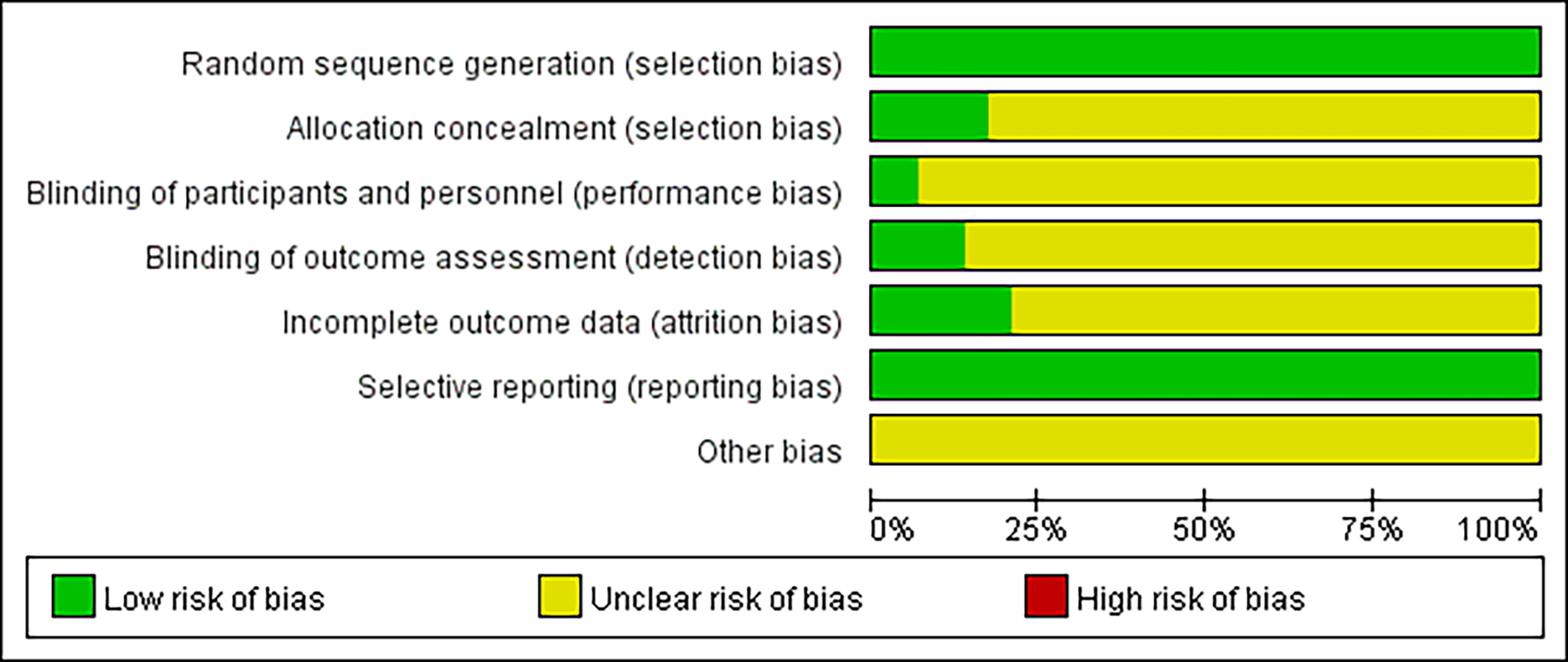

Supplement: Supplementary Figure 1 — Quality assessment percentage graph. [file Image_1.TIF]

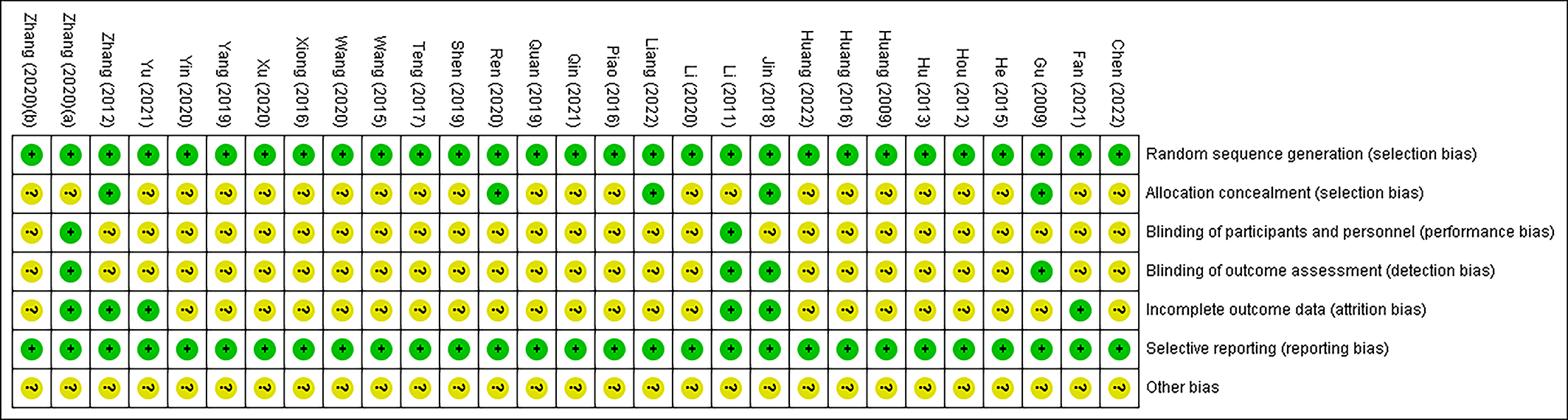

Supplement: Supplementary Figure 2 — Summary chart of quality assessment. [file Image_2.TIF]

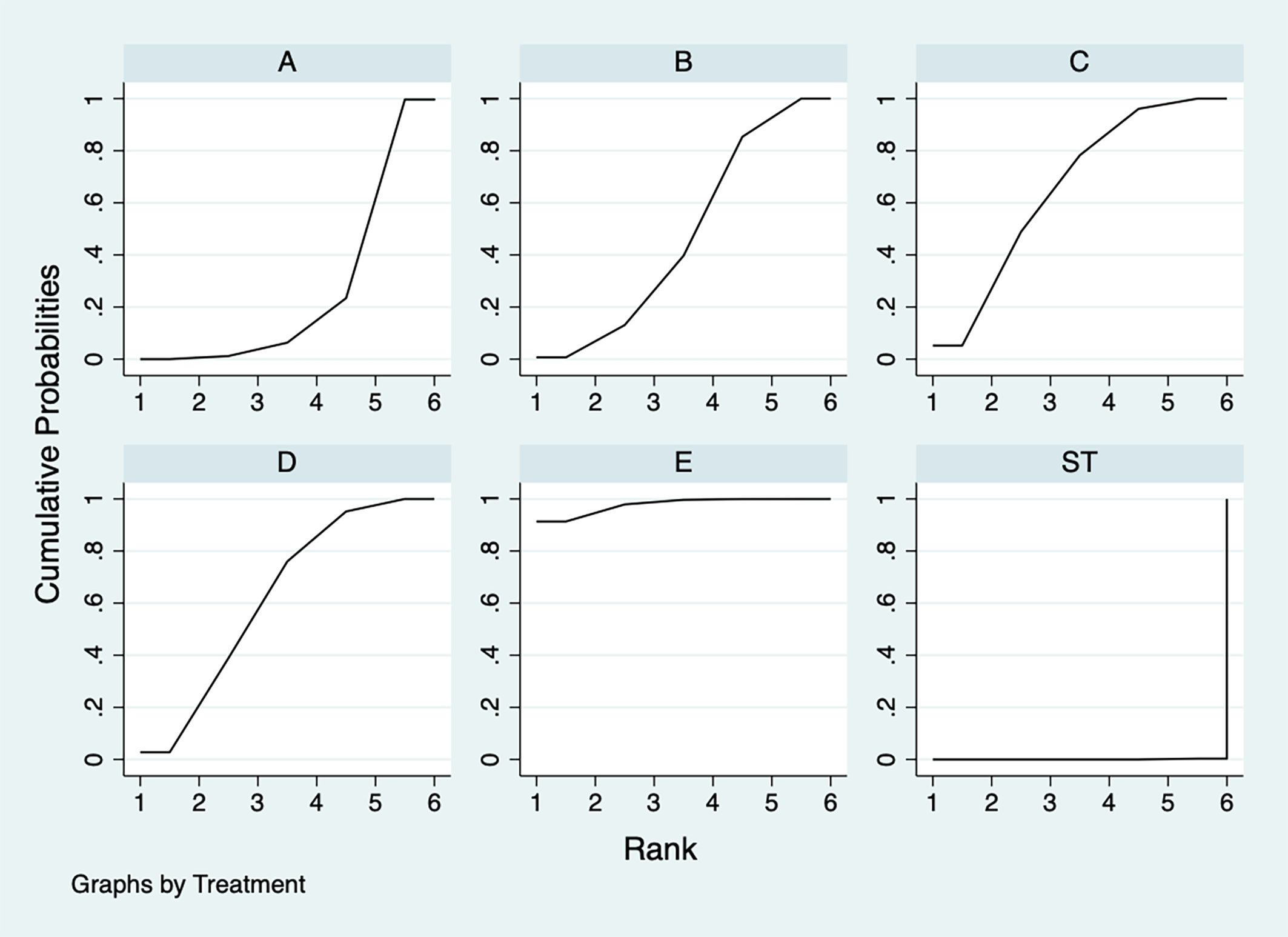

Supplement: Supplementary Figure 3 — Probability ranking results of CER of different interventions. ST, Speech training; BA, body acupuncture; SA, scalp acupuncture; TA, tongue acupuncture; SBA, scalp-body acupuncture; STA, scalp-tongue acupuncture. [file Image_3.TIF]

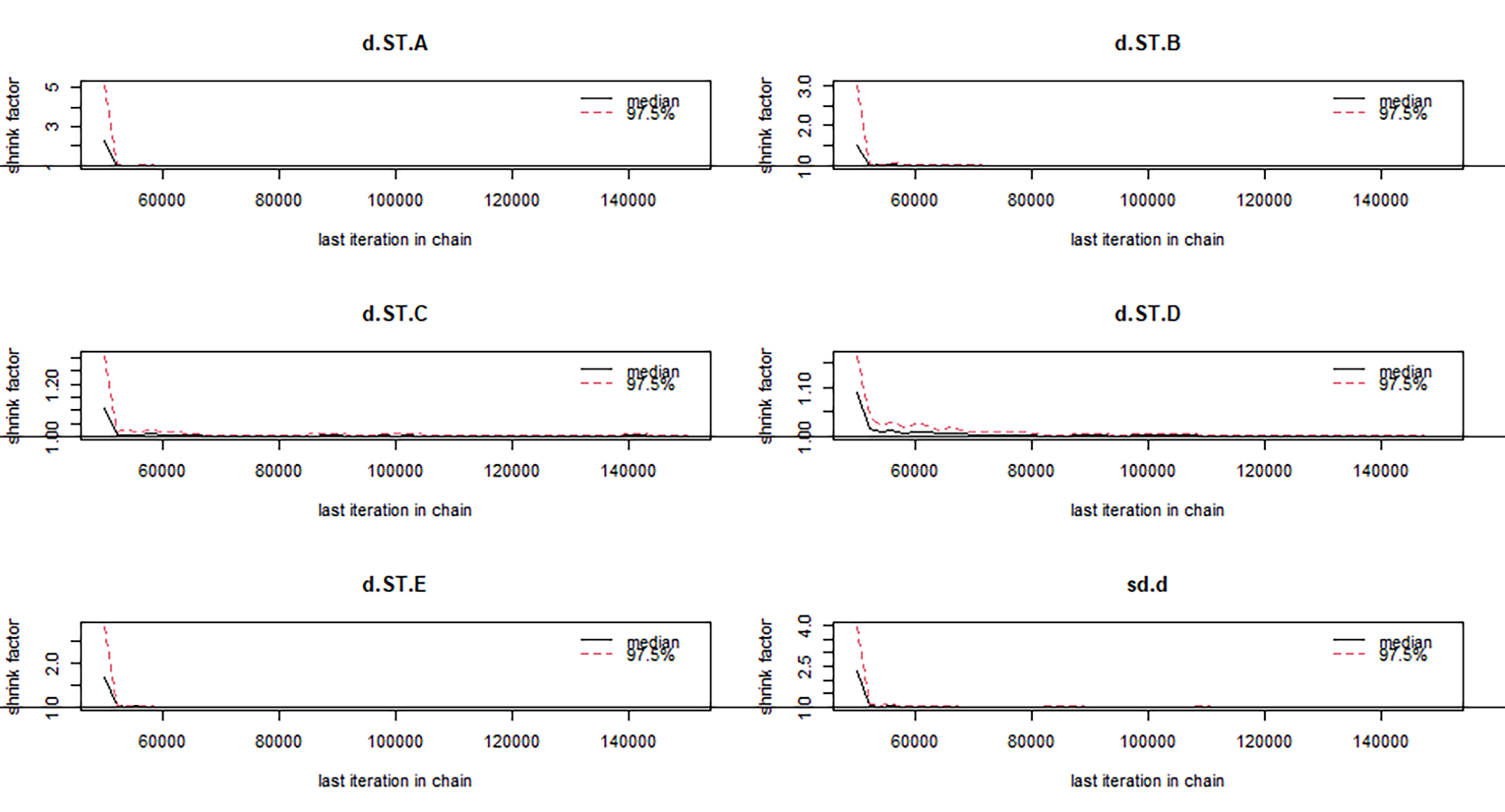

Supplement: Supplementary Figure 4 — PSRF diagnostic diagram for CER. [file Image_4.TIF]

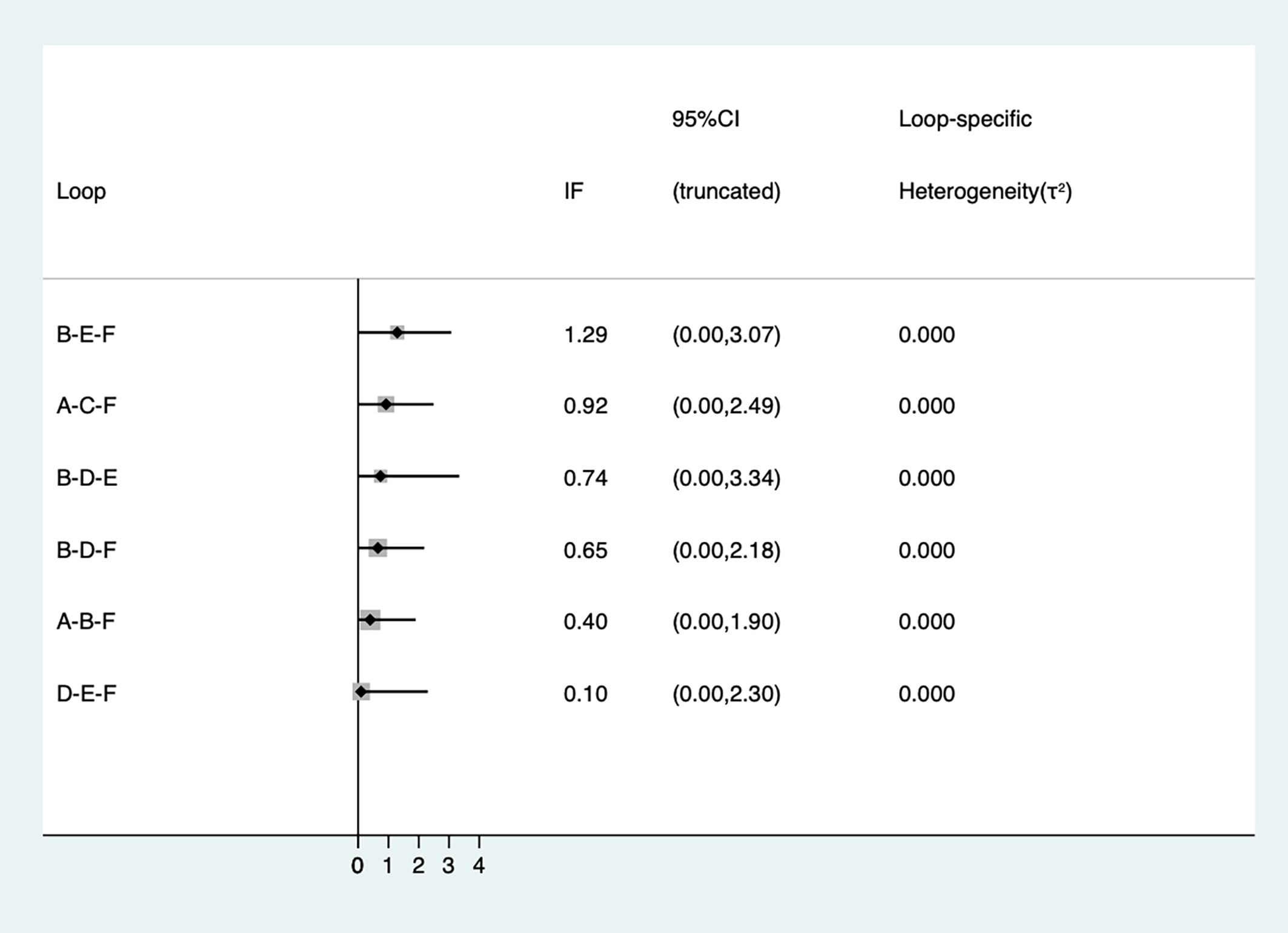

Supplement: Supplementary Figure 5 — Loop inconsistency tests. ST, Speech training; BA, body acupuncture; SA, scalp acupuncture; TA, tongue acupuncture; SBA, scalp-body acupuncture; STA, scalp-tongue acupuncture. [file Image_5.TIF]
