# Supplementary material for: Comparison of the efficacy of acupuncture-related Therapies for post-stroke motor aphasia: A Bayesian network meta-analysis
Source: Front Neurol. 2022 Dec 20;13:992079. doi: 10.3389/fneur.2022.992079 (PMC9810494; doi:10.3389/fneur.2022.992079)
Supplement: Supplementary file 6 [file Table_1.docx]

Supplementary Material 1. The search strategy for PubMed.

| Number | Search terms |
| --- | --- |
| #1 | “Stroke”[Mesh] OR“Strokes”[Title/Abstract] OR “Cerebrovascular Accident”[Title/Abstract] OR “Cerebrovascular Stroke”[Title/Abstract] OR “Apoplexy”[Title/Abstract] OR “Cerebral Stroke”[Title/Abstract]OR “Vascular Accidents, Brain”[Title/Abstract]OR “CAV”[Title/Abstract]OR “cerebral infarction”[Title/Abstract]OR “Ischemic Stroke”[Title/Abstract]OR “cerebral hemorrhage[Title/Abstract]OR “Hemorrhage Stroke”[Title/Abstract] |
| #2 | “Aphasia, Broca”[Mesh] OR “Motor Aphasia”[Title/Abstract] OR “Dysphasia, Broca's”[Title/Abstract] OR “Expressive Aphasia” [Title/Abstract] |
| #3 | “Acupuncture”[Mesh] OR “Pharmacopuncture” [Title/Abstract] OR “Acupuncture Treatment”[Title/Abstract] OR“needling”[Title/Abstract] OR “electric- acupuncture”[Title/Abstract] OR “scalp acupuncture”[Title/Abstract]OR “tongue acupuncture”[Title/Abstract] OR “eye-acupuncture”[Title/Abstract] OR “blood pricking therapy”[Title/Abstract] OR “ear acupuncture”[Title/Abstract] |
| #4 | “Speech training” [Title/Abstract] OR “Speech therapy” [Title/Abstract] OR “Language rehabilitation training”[Title/Abstract] |
| #5 | “Randomized Controlled Trial[Publication Type]OR “RCT randomized controlled”[Publication Type] OR “random allocation”[Title/Abstract] OR “allocation, random”[Title/Abstract] OR “randomized, controlled”[Title/Abstract] OR “clinical trial”[Title/Abstract] |
| #6 | #1 AND #2 AND #3 AND #4 AND #5 |
