# Supplementary material for: Comparison of the efficacy of acupuncture-related Therapies for post-stroke motor aphasia: A Bayesian network meta-analysis
Source: Front Neurol. 2022 Dec 20;13:992079. doi: 10.3389/fneur.2022.992079 (PMC9810494; doi:10.3389/fneur.2022.992079)
Supplement: Supplementary file 7 [file Table_2.docx]

Supplementary Material Table 2. Egger's test for CER

Std_Eff | Coef. Std. Err. t P>|t| [95% Conf. Interval]

slope | -.00506 .1889168 -0.03 0.979 -.3926853 .3825653

bias | .534122 .5349339 1.00  **0.327**  -.5634717 1.631716
